# Novel Furocoumarin Derivatives Stimulate Melanogenesis in B16 Melanoma Cells by Up-Regulation of MITF and TYR Family via Akt/GSK3β/β-Catenin Signaling Pathways

**DOI:** 10.3390/ijms19030746

**Published:** 2018-03-06

**Authors:** Chao Niu, Li Yin, Haji Akber Aisa

**Affiliations:** 1 Key Laboratory of Plant Resources and Chemistry of Arid Zone, State Key Laboratory Basis of Xinjiang Indigenous Medicinal Plants Resource Utilization, Xinjiang Technical Institute of Physics and Chemistry, Chinese Academy of Sciences, Urumqi 830011, China; niuchao@ms.xjb.ac.cn (C.N.); jinshiyi2003@163.com (L.Y.); 2 University of Chinese Academy of Sciences, Beijing 101408, China

**Keywords:** vitiligo, furocoumarin, melanogenesis, SAR, Akt/GSK3β/β-catenin signaling pathways

## Abstract

The extracts of *Ficuscarica* L. and *Psoralen corylifolia* L. are traditional Uygur medicines for the treatment of vitiligo, and its active ingredients furocoumarins, were are found to be the most effective agents against this skin disorder nowadays. Therefore, a series of novel easter derivatives (**8a**–**8p**) of furocoumarin were designed and synthesized based on our previous research to improve this activity in the present study. The synthesized derivatives were biologically evaluated for melanin synthesis in murine B16 cells and the SAR (structure-activity relationship) was summarized. Eight derivatives were more potent than positive control (8-MOP, 8-methoxypsoralan), especially compounds **8n** (200%) and **8o** (197%), which were nearly 1.5-fold potency when compared with 8-MOP (136%). Furthermore, the signaling pathway by which **8n** activates the melanin biosynthesis was defined. Our results showed that it not only elevated the melanin content, but also stimulated the activity of tyrosinasein a concentration-dependent manner. Increasing of phosphorylation of Akt (also named PKB, protein kinase B) and non-activated GSK3β (glycogen synthase kinase 3 beta), which inhibited the degradation of β-catenin were observed through Western blot analysis. The accumulation of β-catenin probably led to the activation of transcription of MITF (microphthalmia-associated transcription factor) and TYR (tyrosinase) family, as well as the subsequent induction of melanin synthesis.

## 1. Introduction

Vitiligo also named leukoderma, is an autoimmune disease that results in prominent white patches of the skin [1]. It can involve any part of the body where melanocytes resided and cause both functional and physiological abnormalities in the affected skin. Many possible causes of vitiligo have been proposed including immunologic, genetic, stress, neural mechanism, and biochemical factors [2]. However, it is believed that the disease is mainly caused by destruction of the melanocyte and obstruction of the melanin synthesis [3,4].

Melanin, derived from dopaquinone and synthesized in the melanosomes of melanocytes, serves a number of valuable physiological functions [5]. The melanin biosynthesis is regulated by enzymatic cascade, such as tyrosinase, tyrosinase-related protein 1 (TRP-1) and tyrosinase-related protein 2 (TRP-2) [6]. Among them, tyrosinase is regarded as the rate-limiting enzyme of process, which modulates this process by catalyzing the hydroxylation of tyrosine into 3,4-dihydroxyphenylalanine (DOPA) and the further oxidation of DOPA into dopaquinone [7,8].

**Figure 1:**
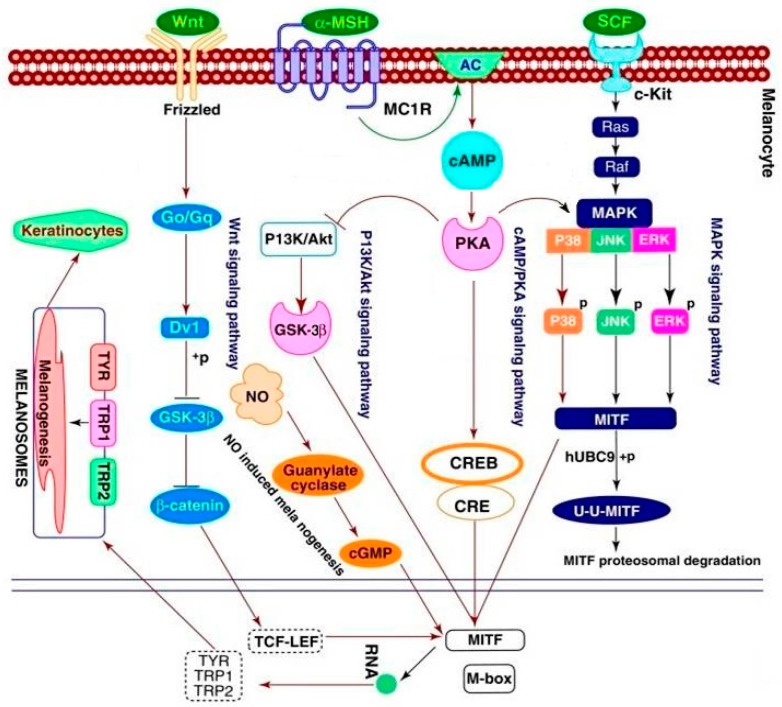
Regulation of melanogenesis through different signaling pathways [10]. α-MSH (α-melanocyte-stimulating hormone), SCF (stem cell factor), AC (adenylate cyclase), MC1R (melanocortin 1 receptor), cAMP (cyclic Adenosine monophosphate), PKA (protein kinase A), MAPK (mitogen-activated protein kinase), GSK (glycogen synthase kinase), JNK (c-Jun N-terminal kinase),ERK (extracellular regulated protein kinases), TYR (tyrosinase), TRP1 (Tyrosinase-related protein 1), TRP2 (Tyrosinase-related protein 2) CREB (cAMP-response element binding protein), CRE (cAMP response element), MITF (microphthalmia-associated transcription factor), TCF-LEF (T cell factor/lymphoid-enhancing factor-1).

Several signaling pathways are presented to clarify the specific mechanism controlling melanin biosynthesis via tyrosinase family, as described in Figure 1 [9,10]. MITF, a master regulator of melanogenesis that is involved in these pathways, upregulates the melanogenesis enzymes TYR, TRP-1 and TRP-2 via binding to the M-box motif in their promoter regions [11]. In addition, MITF modulates melanocyte function including melanocyte differentiation, pigmentation, proliferation, and cell survival [12].

**Figure 2:**
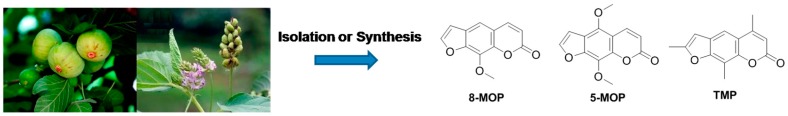
The structures of furocoumarins (psoralens) isolated from the plant *Ficuscarica* L. and *Psoralen corylifolia* L. or synthesized [13]. 5-MOP (5-methoxypsoralan), TMP (trimethylpsoralen).

The extract of *Ficuscarica* L. and *Psoralen corylifolia* L. (Figure 2) [13,14] alone or in combination are popular Uygur medicines that are used for vitiligo in Xinjiang and other Central Asian countries hundreds of years ago [15,16]. During the last century, several furocoumarins (psoralens), such as 8-methoxypsoralen (8-MOP), 5-methoxypsoralen (5-MOP), and 4,5,8-trimethylpsoralen (TMP) were isolated from the plants or totally synthesized [17,18]. These compounds were proved to show strong photosensitivity [19] later, which may be used for the treatment of vitiligo with subsequent exposure to long-waved ultraviolet radiation [20,21]. Although PUVA (psoralens + UVA) (ultraviolet radiation A) [22] was accompanied with some undesired side effects [23,24,25], the therapy is still the most successful (or less disappointing) one for skin repigmentation today. Unfortunately, the precise mechanism of PUVA in the treatment of vitiligo is obscure, let al.one the target of these drugs.

However, few furocoumarin derivatives and analogues with potential anti-vitiligo activity were reported in spite of their high efficiency against the disease. Our group had been dedicated on the drug development of the vitiligo for many years [26,27,28,29,30,31]. In our previous research, a great augumentation to the melanin synthesis was observed when aromatic groups were introduced to C-5 position of the furocoumarin derivative, which suggested that more structural modification should be pursued on this position to search for novel bioactive molecules on pigmentation that may be developed as better medication for the vitiligo.

Therefore, sixteen ester derivatives (**8a**–**8p**) of furocoumarin were prepared, then submitted to the activity assay of melanogenesis in B16 cells and the SAR was summarized as well. Furthermore, the tyrosinase activity and expression of proteins related to melanin biosynthesis were determined by Western blot analysis in cells treated with the most promising derivative (**8n**) for understanding the mechanism underlying the observed effect.

## 2. Results and Discussion

### 2.1. Synthesis

**Scheme 1:**
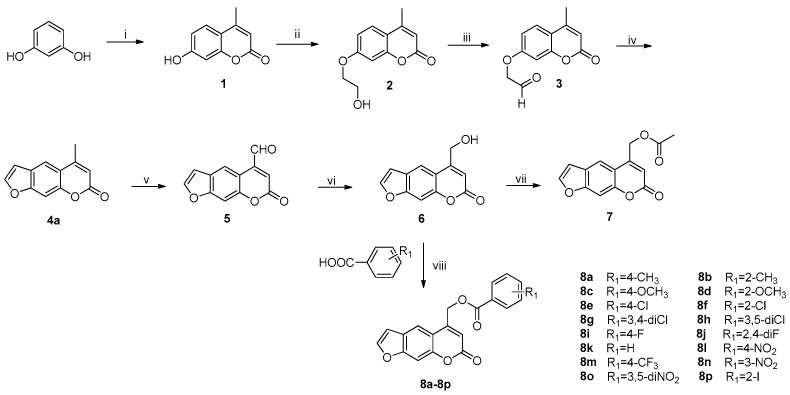
Synthetic route for the furocoumarin derivatives (i) ethyl acetoacetate, H_2_SO_4_, 60°C; (ii) chloroethanol, K_2_CO_3_, acetone, reflux; (iii) −78°C, oxalyl chloride, DMSO (dimethyl sulfoxide), triethylamine, DCM (dichloromethane); (iv) 1M NaOH aqueous solution, 1,4-dioxane; (v) SeO_2_, xylene, reflux; and, (vi) NaBH_4_, ethanol, rt; (vii) pyridine, Ac_2_O, rt. (vii) DCC (dicyclohexylcarbodiimide), DMAP (4-dimethylaminopyridine), DCM, 0°C-rt (room temperature).

The synthetic route of the target compounds was described in Scheme 1. The intermediate **1** (4-methylumbelliferone) prepared from resorcinol via Pechmann reaction [32,33], was converted to compound **2** by Williamson reaction refluxing with chloroethanol in the presence of anhydrous K_2_CO_3_. Compound **2** was further oxidized to aldehyde **3** at −78 °C by an optimized Swern oxidation in excellent yields. Intermolecular cycloaddition of the intermediate **3** yielded compound **4a** in the presence of 1M NaOH. It is notable that a mixed solvent (*V*_water_:*V*_1,4-dioxane_ = 1:1) was applied to get a homogeneous solution of aldehyde **3** for the easy cycloaddition.

Selenium dioxide was applied in selective oxidation of compound **4a** to produce aldehyde **5**. Compound **5** was reduced with NaBH_4_ to achieve alcohol **6 [34,35]**, which was further esterified to give **7** and **8a**–**8p** under different conditions, respectively [32,36].

**Figure 3:**
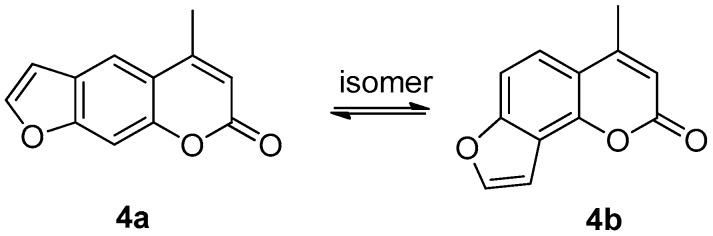
Structure of psoralen **4a** and angelicin **4b**.

The oxidation of intermediate **2** to **3** was the crucial step in the preparation of compounds **4a**, which bearing no substituent group on the furan ring. With the application of DMSO-(COCl)_2_ oxidation system, compound **2** was smoothly transformed to aldehyde **3** in almost quantitative yield as we did in our previous work. In the cyclization of intermediate **3**, two sets of compounds (psoralen: **4a** and angelicin: **4b**) were produced in one step, which was isomer to each other (Figure 3). The yield of **4a** in the mixture was much higher than angelicin (**4b**) under basic condition.

### 2.2. Melanin Synthesis Evaluation of ***8a**–**8p***

**Figure 4:**
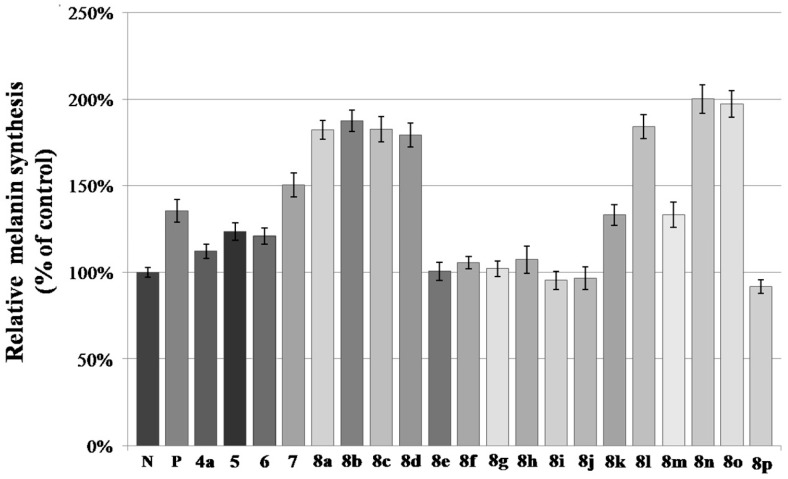
Stimulation of melanin content of B16 cells by furocoumarin derivatives. N means negative control; P means positive control (8-MOP); The B16 cells were treated with 50 µM of different furocoumarin derivatives for 48 h. After that, melanin content was measured directly. Values are expressed as the mean ± SD of three separate experiments.

**Figure 5:**
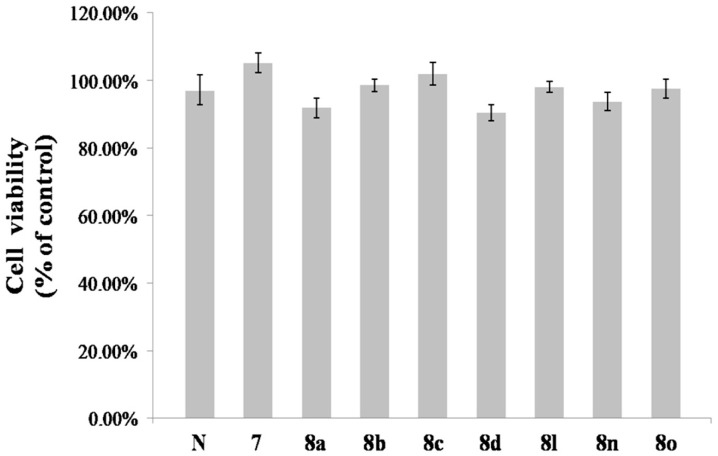
Effect of furocoumarin derivatives on B16 cells viability. N means negative control; The B16 cells were incubated with 50 µM of different furocoumarin derivatives for 48 h and the cell viability was assayed by adding CCK-8 (Cell Counting Kit) solution. Values are expressed as the mean ± SD of three separate experiments.

All of the synthesized compounds were screened for their activity on melanin synthesis in murine B16 cells, with a known method (Figure 4) [37]. In order to avoid the possibility that inhibition of melanin synthesis was due to cytotoxicity, we performed CCK-8 assay as well to determine whether these active ones were cytotoxic to B16 cells. The result showed that the cells treated with compounds for 48 h caused little cytotoxicity when compared with the control at the dosage of 50 µM (Figure 5).

According to the result (Figure 4), half of the tested compounds (**7**, **8a**–**8d**, **8l**, **8n**–**8o**) exhibited a stronger activity than the positive control (8-MOP) with a value from 182% to 200%. For compounds **6** and **7**, further esterification of hydroxymethyl with acetic anhydride caused an increase in activity.

The activity of ester derivatives, was which substituted by halogens, decreased dramatically (**8e**–**8j**, **8p**) as compared with **8k**, especially for the ones bearing F and I, regardless of the number and position of halogens on benzene. Compounds with -CH_3_ (**8a**–**8b**) and -OCH_3_ (**8c**–**8d**) on *ortho-* and *para-* position of benzene showed a much higher activity than the halogenated ones, which was consistent with our previous findings [28]. The interesting thing was that the strong electron-withdrawing group (EWG), such as -NO_2_, may be favorable to promote the melanin content, especially for **8n** (200%) and **8o** (197%), which demonstrated the best activity of these derivatives. It seemed that this effect had no relationship with the position and number of the -NO_2_ on benzene.

Overall, both EDG (electron-donating group) (-CH_3_, -OCH_3_) and EWG (-NO_2_) on benzene greatly improved the melanin biosynthesis, which indicated that the electrostatic interaction between derivatives and receptor protein may have little correlation with activity.

### 2.3. Effect of ***8n*** on B16 Melanoma Cells Viability

**Figure 6:**
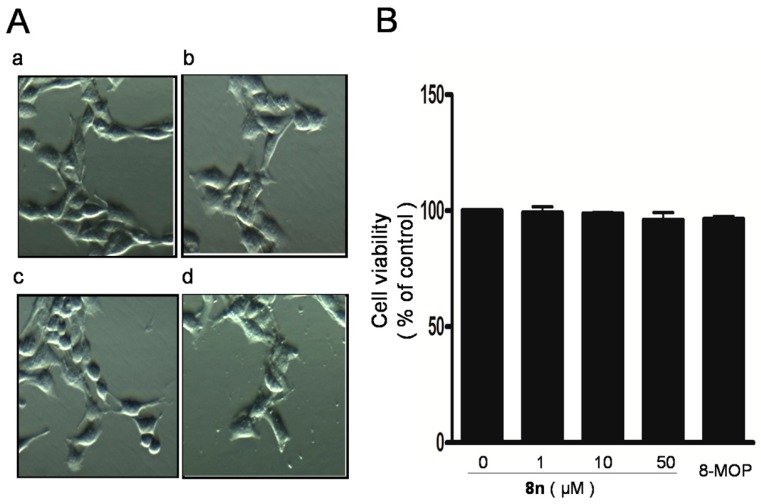
(**A**(**a**–**d**)) Effects of **8n** on cell morphology. B16 cells were treated with 0.1% DMSO (dimethyl sulfoxide) as vehicle (**a**) or with **8n** at 1 (**b**), 10 (**c**) and 50 μM (**d**) for 48 h. Cell morphology was observed under a microscope. Magnification, ×200. (**B**) Effects of **8n** on B16 melanoma cell viability. B16 cells were treated with 50 μM 8-MOP as post control and with **8n** at concentrations of 0–50 μM for 48 h. Cell viability was measured by Cell Counting Kit-8 assays. The data are shown as the mean ± SD; *n* = 3.

The murine melanoma B16 cells were treated with **8n** at concentrations of 0–50 μM for 24 h and examined the cytotoxicity by CCK-8 assay. As shown in Figure 6A(a–d), compound **8n** did not induce any change in cell morphology when compared with untreated cells and showed no increase in cytotoxicity (Figure 6B). Accordingly, 0–50 μM **8n** was applied in the subsequent experiments.

### 2.4. Effect of ***8n*** on Melanogenesis and Tyrosinase in B16 Melanoma Cells

**Figure 7:**
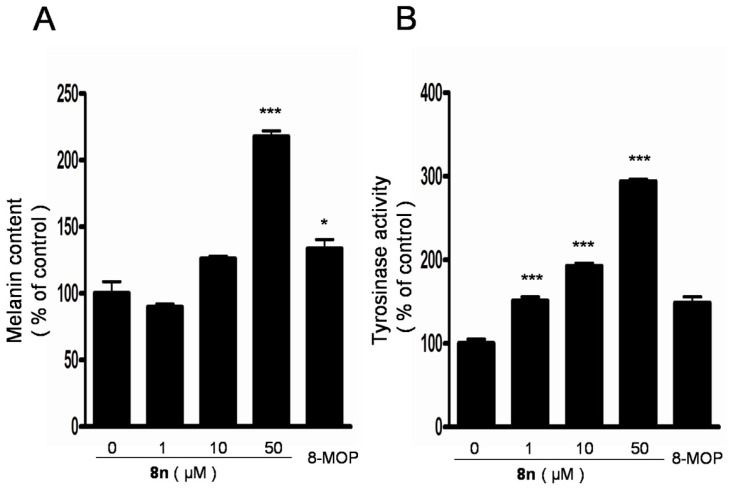
(**A**,**B**) Effects of **8n** on melanogenesis and tyrosinase in B16 melanoma cells. Cells were treated with 0.1% DMSO as vehicle or with **8n** at 1, 10, 50 μM, and 50 μM 8-MOP as positive control for 48 h or 24 h to evaluate melanin content and tyrosinase activity, respectively. Each percentage value for treated cells is reported relative to that of 0.1% DMSO cells. The data are shown as the mean ± SD; *n* = 3, * *p* < 0.05, *** *p* < 0.001 as compared with 0.1% DMSO cells.

The melanin production was measured in B16 melanoma cells after 48 h of treatment with **8n** at 0–50 μM. As shown in Figure 7A, treatment with **8n** improved the melanin synthesis in a dose-dependent manner, and the melanin content increased 63% compared with 8-MOP at 50 μM. (0 μM, 100 ± 15.4%; 1 μM, 89.7 ± 4.0%; 10 μM, 125.9 ± 3.7%; 50 μM, 217.6 ± 7.5%; 8-MOP, 50 μM, 133.5 ± 12.1%). After that, the tyrosinase activity in B16 cells was studied as well and it showed a similar increasing trend in response to **8n** treatment in Figure 7B. Specifically, when compared with 8-MOP treated cells, treatment with compound at a concentration of 50 μM resulted in an approximately 98% activation of intracellular tyrosinase in the B16 melanoma cells.(0 μM, 100 ± 9.2%; 1 μM, 150.7 ± 8.7%; 10 μM, 192.4 ± 6.4%; 50 μM, 293.9 ± 4.3%; 8-MOP, 50 μM, 148.4 ± 12.9%).

### 2.5. Effects of ***8n*** on TYR Family and MITF Expression Levels in B16 Melanoma Cells

**Figure 8:**
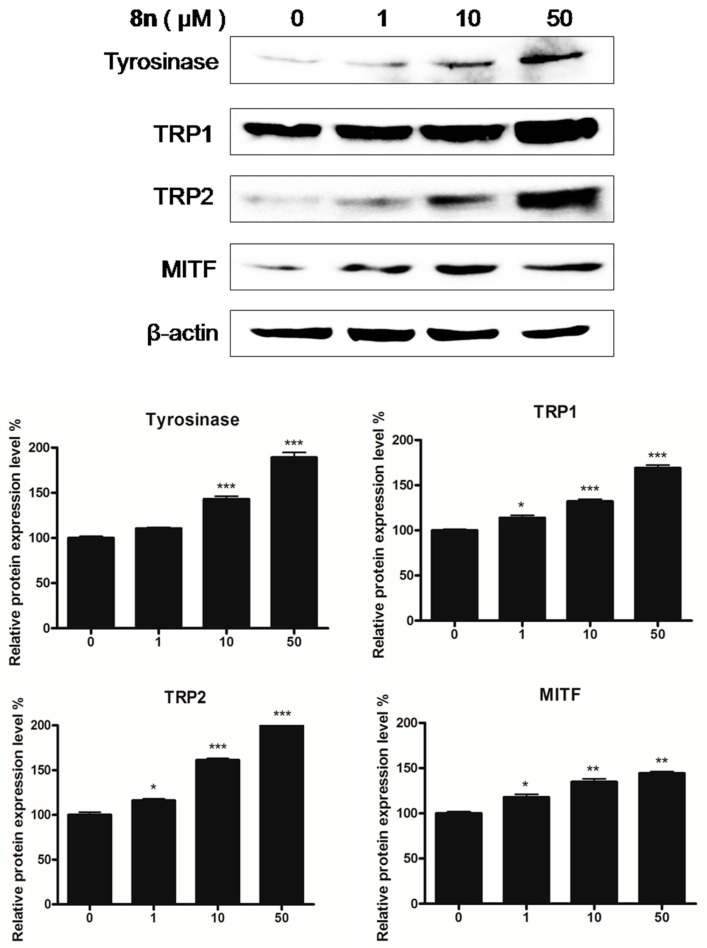
Representative western blots illustrating expression of TRPs and MITF. B16 cells were treated with **8n** at 0, 1, 10 and 50 µM for 48 h. Tyrosinase, TRP1, TRP2, and MITF protein expression were detected by Western blotting. Results were normalized against β-actin expression; Densitometric scanning of band intensities obtained from three individual experiments was used to quantify change of protein expression (control value taken as one-fold in each case). The Data are shown as mean ± SD of three independent experiments. * *p* < 0.05, ** *p* < 0.01, *** *p* < 0.001 vs. controls.

In order to clarify the mechanism that is responsible for the elevation in pigmentation, the expression levels of TYR family and MITF were examined by Western blot. B16 melanoma cells were treated with various concentrations of **8n** (0–50 μM) for 48 h. As shown in Figure 8, the tyrosinase, TRP-1, and TRP-2 levels markedly increased following treatment with **8n**. As the TYR family was the downstream genes of MITF, the level of MITF was measured, and MITF expression exhibited comparative changes. These results suggested that **8n** induced melanin synthesis through the up-regulation of the TYR family and MITF at protein levels.

### 2.6. Effect of ***8n*** on Akt and Wnt/β-Catenin Signaling Pathways Involved Inmelanogenesis

**Figure 9:**
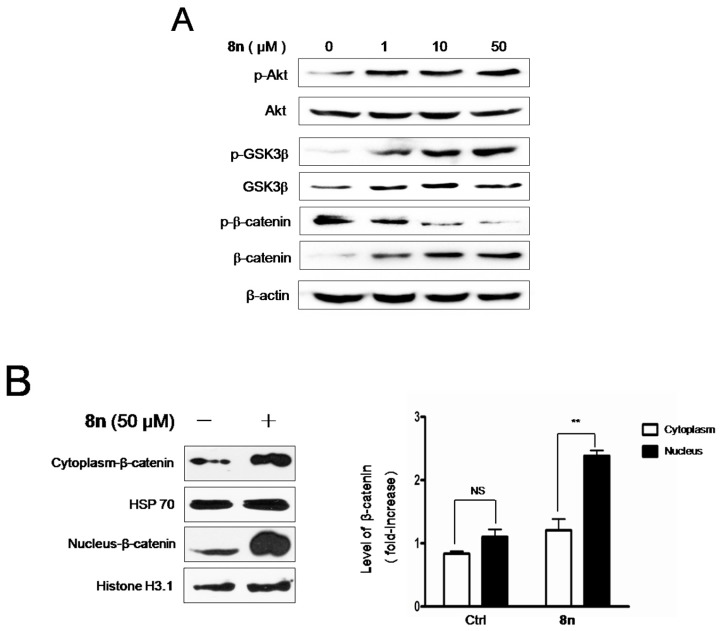

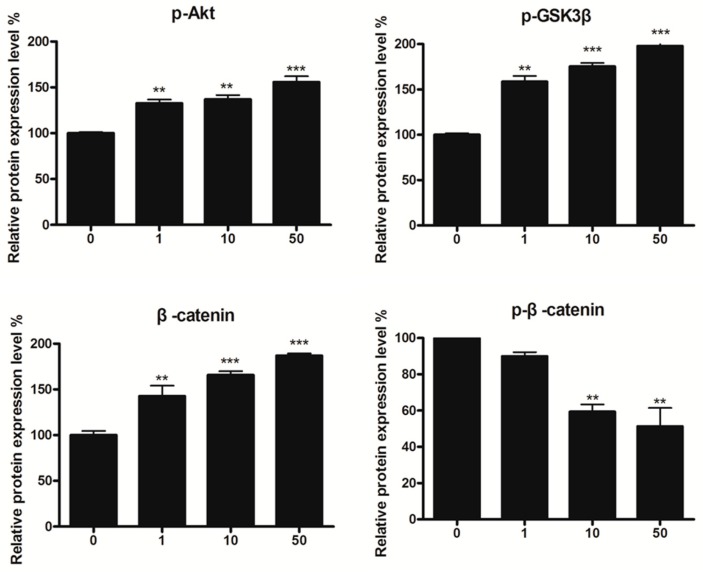
Effect of **8n** on Akt and Wnt/β-catenin signaling pathways involved in melanogenesis. (**A**) B16 cells were treated with **8n** at the 0, 1, 10, and 50 μM for 48 h, the expression of Akt, GSK3β and β-catenin were measured by Western blot. (**B**) B16 cells were treated with **8n** of 50 μM for 12 h, the expression levels of proteins including β-catenin in cytoplasm and nucleus were detected by using Western blot; Densitometric scanning of band intensities obtained from three individual experiments was used to quantify change of protein expression (control value taken as one-fold in each case). The Data are shown as mean ± SD of three independent experiments. * *p* < 0.05, ** *p* < 0.01, *** *p* < 0.001 vs. controls.

The Wnt signaling pathway was demonstrated as one of the signaling processes in melanin synthesis [38,39]. In order to determine the molecular mechanism of the superior pigmented effect of **8n**, Western blot analysis for the changes of β-catenin was introduced. As shown in Figure 9A, the phosphorylation of β-catenin at Ser33, decreased with a concomitant increase of total β-catenin induced by **8n**. Moreover, β-catenin content in the nucleus (the active β-catenin) enhanced obviously after 12 h of **8n** treatment as compared with untreated cells, while the content in the cytoplasm did not change (Figure 9B).

**Figure 10:**
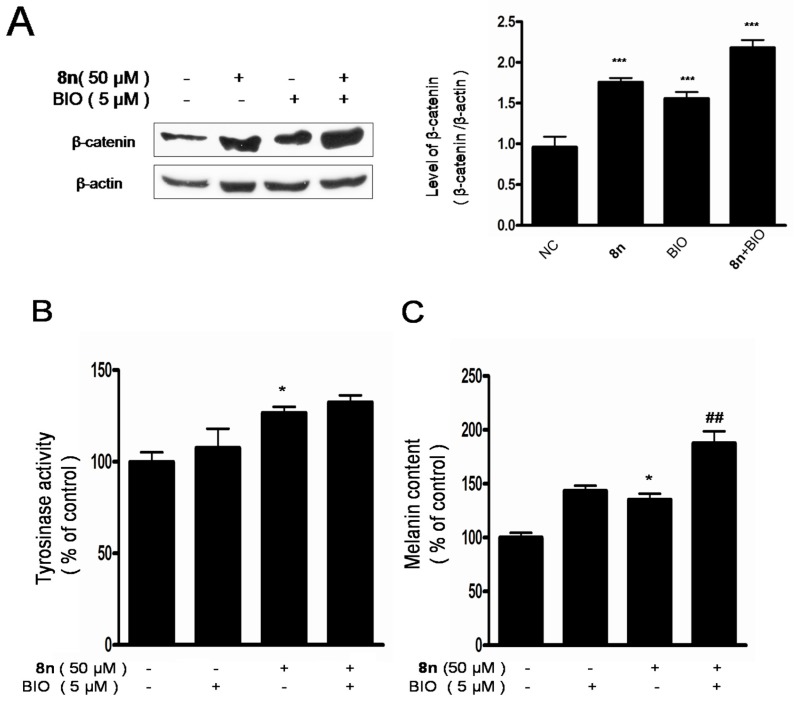

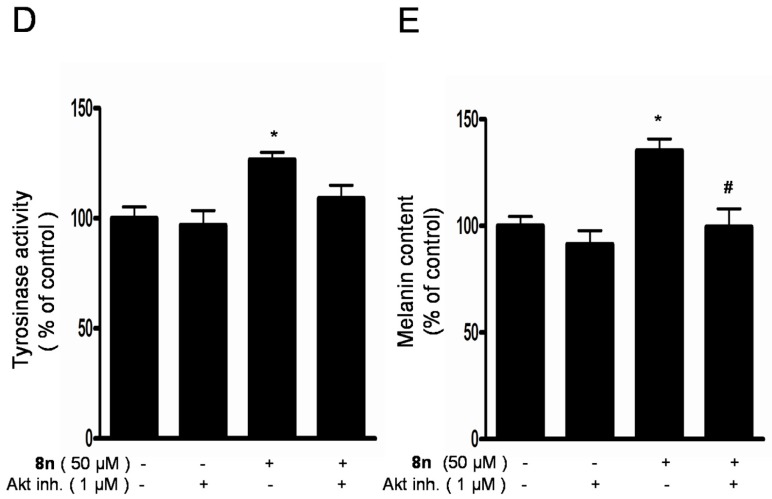
(**A**) β-catenin was detected in B16 cells pre-treated with or without BIO (5 μM) and incubated with **8n** (50 μM) or not. (**B**–**E**) Inhibitors (BIO 5 μM, Akt inhibitor IV 1 μM) were pre-incubated with B16 cells for 2 h before addition of **8n** at 50 μM, followed by an additional incubation for 24 h or 48 h for tyrosinase activity and melanin content, respectively. * *p* < 0.05compared with control; ^#^ *p* < 0.05, ^##^ *p* < 0.01 as compared with **8n** stimulation. “-” means pre-treated without **8n** or Bio or Akt; “+” means pre-treated with **8n** or Bio or Akt.

GSK3β, which is a negative regulator of Wnt signaling, could phosphorylate the residues of β-catenin, which contributes to the degradation of β-catenin through proteasome [40]. As expected, the level of phosphorylation of GSK3β rose up in B16 cells treated with **8n** (Figure 9A). BIO (6-bromoindirubin-3-oxime) was able to inhibit the phosphorylation of GSK3β and regarded as a selective GSK3β inhibitor [41]. The graph revealed that the BIO increased the accumulation of β-catenin content induced by **8n** (Figure 10A). Being consistent with this, it also improved the TYR activity and melanin content in B16 cells in the presence of **8n** evidently (Figure 10B,C).

Currently, there are numerous reports about the close relationship between PI3K/Akt (phosphatidylinositide 3-kinases/protein kinase B) signal pathway and GSK3β [42,43,44,45,46]. Activated Akt can phosphorylate GSK3β resulting in inactivating GSK3β and inhibit the degradation of β-catenin [47]. Our data indicated that content of p-Akt and p-GSK3β increased in B16 cells in the presence of **8n** after 48 h (Figure 9A). However, co-treatment with Akt inhibitor IV (5-(2-benzothiazolyl)-3-ethyl-2-[2-(methylphenylamino)ethenyl]-1-phenyl-1*H*-benzimidazolium iodide, a selective inhibitor of Akt that decreased the phosphorylation level of Akt and inhibited PI3K/Akt signal pathway [48]) distinctly aborted **8n**-mediated tyrosinase activity and melanin content (Figure 10D,E).

These results suggested that **8n** exerts a pigmented effect through the activation Wnt/β-catenin signaling pathway by regulating Akt signal molecule.

The results of the present study indicated that 50 μM of **8n** was more effective than the melanogenesis agent (8-MOP) since it increased melanin content (Figure 7A) and tyrosinase activity better than the latter (Figure 7B). In addition, the compound significantly up-regulated the level of MITF, and its related proteins, including TYR, TRP1, and TRP2, in a dose-dependent manner. Taken together, these findings suggested that **8n** was an effective tyrosinase activator to promote the melanin production in B16 cells.

According to the literature, the Wnt signalling pathway played a pivotal role in melanogenesis. By phosphatidylinositol-3-kinase (PI3K)/Akt activation and GSK3β phosphorylation, MITF binded to the M-box of the tyrosinase promoter, thereby up-regulated melanogenic protein expression and induced melanogenesis [49,50,51]. Western blot analysis proved **8n** could activate the phosphorylation of Akt and GSK-3β, indicating that it raised MITF transactivation of tyrosinase through GSK-3β, which was similarly as mentioned in previous study [52]. Moreover, it was found that pretreatment with BIO enhanced **8n**-induced melanin production and tyrosinase activation (Figure 10B,C) when the up-regulation of melanin synthesis by the compound regulated by Akt/GSK3β/β-catenin signaling was confirmed. However, co-treatment with Akt inhibitor IV significantly reversed these results (Figure 10D,E). Thus, Melanin production mediated by compound **8n** was probably triggered through both Akt and Wnt pathways, consistent with reports revealing that activation of Akt/GSK3β/β-catenin influenced melanin production in B16 melanoma cells [53].

## 3. Materials and Methods

### 3.1. Chemistry

Reagents and solvents were purchased from Sigma (Shanghai, China), and used without further purification. Thin-layer chromatography (TLC) was carried out on glass plates coated with silica gel (Qingdao Haiyang Chemical Co., Qingdao, China, G60_F-254_) and visualized by UV light (254 nm). The products were purified by column chromatography over silica gel (Qingdao Haiyang Chemical Co., 200–300 mesh). Melting points were determined on a Buchi B-540 apparatus and uncorrected. All of the NMR (nuclear magnetic resonance) spectra were recorded with a Varian400, 600 MHz NMR spectrometer in CDCl_3_, using TMS (tetramethylsilane) as an internal standard. High-resolution mass spectra (HRMS) were recorded on AB SCIEX QSTAR Elite quadrupole time-of-flight mass spectrometry. The IR (infrared spectroscopy) data were recorded on a Thermo Fisher Scientific Nilolet 6700 FT-IR infrared spectrometer (KBr).

#### 3.1.1. Preparation of 4-Methylumbelliferone (**1**)

To an ice-cold solution of resorcinol (2.0 g, 18.2 mol) in dioxane, conc. H_2_SO_4_ (0.5 mL) was added dropwise under 20 °C. After the addition of conc. H_2_SO_4_, ethyl acetoacetate (2.8 g, 21.8 mmol) was added, and the mixture was heated to 60 °C for 4 h. Then, the mixture was poured into cold water, and the precipitate was filtered and dried under reduced pressure. The resulting mixture was recrystallized from methanol to give compound as white needle crystals. Yield 92%, m.p. 202–204 °C.

#### 3.1.2. Preparation of 7-(2-Hydroxyethoxy)-4-methyl-*2H*-chromen-2-one (**2**)

A mixture of **1** (0.88 g, 5.0 mmol) with chloroethanol (0.60 g, 7.5 mmol) and anhydrous K_2_CO_3_ (1.4 g, 10 mmol) in acetone (50 mL) was refluxed under stirring for 4 h. After cooling, the reaction mixture was filtered, and the filtrate was evaporated under reduced pressure. The obtained residue was purified by silica gel chromatography with petroleumether/ethylacetate or chloroform to give intermediate **3**. Yield 95%, white solid, m.p. 108–110 °C; ^1^H NMR (400 MHz, CDCl_3_) *δ*7.51 (d, *J* = 9.0 Hz, 1H), 6.91–6.83 (m, 2H),6.15 (d, *J* = 1.1 Hz, 1H), 4.15 (t, *J* = 8.7 Hz, 2H), 4.01 (m, 2H), 2.40 (d, *J* = 1.1 Hz, 3H).

#### 3.1.3. Preparation of 7-(2-Oxoethoxy)-4-methyl-*2H*-chromen-2-one (**3**)

A solution of oxalyl chloride (0.252 g, 2 mmol) in anhydrous CH_2_Cl_2_ (5 mL) was cooled to −78 °C under a nitrogen atmosphere, and a solution of DMSO (0.156 g, 2 mmol) in CH_2_Cl_2_ (2 mL) was added dropwise (the temperature was kept below −65 °C), and stirred for 10 min. A solution of alcohols **2** (0.22 g, 1 mmol) in CH_2_Cl_2_ (15 mL) was added dropwise at a temperature below –65 °C and stirred for another 30 min. Triethylamine (0.505 g, 5 mmol) was then added dropwise. The reaction mixture was stirred for 15 min at a temperature below −65 °C and allowed to warm up to room temperature. The reaction mixture was diluted with CH_2_Cl_2_ and was filtered through a pad of silica gel. Removal of the solvent under vacuum afforded the aldehydes **3**. Yield 97%, white solid, m.p. 140–142 °C; ^1^H NMR (400 MHz, CDCl_3_) *δ*9.85 (s, 1H), 7.53 (d, *J* = 8.8 Hz, 1H), 6.89 (dd, *J* = 8.8, 2.6 Hz, 1H), 6.78 (d, *J* = 2.5 Hz, 1H), 6.15 (d, *J* = 1.0 Hz, 1H), 4.68 (s, 2H), 2.39 (d, *J* = 0.9 Hz, 3H).

#### 3.1.4. Preparation of Compounds **4**

A solution of aldehydes **3** (0.5 mmol) in H_2_O-dioxane (1:1, 3 mL) was added dropwise to a refluxing aqueous solution of NaOH (1.0 M, 10 mL) under stirring for more than 30 min. The resulted mixture was stirred at reflux for an additional 4 h and then cooled down to room temperature. The solution was acidified to pH = 3 with 85% phosphoric acid and was left overnight. The resultant mixture was extracted with ethylacetatethree times. The organic phase was then washed with brine and dried over anhydrous Na_2_SO_4_. After removal of the solvents, the residue was purified by flash chromatography on silica gel eluted with *V*_petroleumether_:*V*_ethylacetate_ = 1:10 to afford a pair of isomer.

5-Methyl-*7H*-furo[3,2-g]chromen-7-one (**4a**). Yield 73%, white solid, m.p. 160–162 °C; ^1^H NMR (400 MHz, CDCl_3_) *δ* 7.81 (s, 1H), 7.69 (d, *J* = 2.2 Hz, 1H), 7.47 (s, 1H), 6.85 (d, *J* = 2.1 Hz, 1H), 6.27 (s, 1H), 2.50 (s, 3H). ^13^C NMR (101 MHz, CDCl_3_) *δ* 161.24, 156.43, 152.78, 151.75, 146.96, 124.77, 116.70, 113.62, 106.67, 100.00, 19.30; IR (KBr) *v*: 2921, 1723, 1630, 1385, 1137, 1031, 928 cm^−1^; HRMS (ESI) calcd for C_12_H_9_O_3_[M + H]^+^ 201.0552, found 201.0540.

4-Methyl-*2H*-furo[2,3-h]chromen-2-one (**4b**). Yield 11%, white solid, m.p. 119–120 °C; ^1^H NMR (400 MHz, CDCl_3_) *δ* 7.69 (d, *J* = 2.2 Hz, 1H), 7.53 (d, *J* = 8.8 Hz, 1H), 7.45 (d, *J* = 8.7 Hz, 1H), 7.15 (d, *J* = 2.1 Hz, 1H), 6.28 (s, 1H), 2.51 (s, 3H). ^13^C NMR (101 MHz, CDCl_3_) *δ* 160.89, 157.25, 153.58, 145.76, 120.48, 117.00, 112.86, 110.02, 108.41, 104.37, 19.44; IR (KBr) *v*: 2919, 1718, 1617, 1264, 1065, 761 cm^−1^; HRMS (ESI) calcd for C_12_H_9_O_3_ [M + H]^+^ 201.0552, found 201.0559.

#### 3.1.5. Preparation of 7-Oxo-*7H*-furo[3,2-g]chromene-5-carbaldehyde (**5**)

Powdered SeO_2_ (3.33 g, 30 mmol) was added to a solution of **4a** (8.0 g, 20 mmol) in 20 mL of hot dry xylene and the mixture were refluxed for 12 h with vigorous stirring underthenitrogen. The reaction mixture was filtered to remove black Se, and the deep orange filtrate was allowed to stand overnight. Almost pure crystals of **5** could be separated from the solution. Yield 63%, yellow solid, m.p. 178–180 °C; ^1^H NMR (400 MHz, CDCl_3_) *δ* 10.14 (s, 1H), 8.90 (s, 1H), 7.72 (d, *J* = 2.1 Hz, 1H), 7.54 (s, 1H), 6.89 (d, *J* = 2.0 Hz, 1H), 6.86 (s, 1H).

#### 3.1.6. Preparation of 5-(Hydroxymethyl)-*7H*-furo[3,2-g]chromen-7-one (**6**)

Compound **5** (4.28g, 20 mmol) was dissolved in ethanol (130 mL), sodium borohydride (380 mg, 10.0 mmol) was added, and the solution was stirred for 2 h at room temperature. Thereafter the suspension was carefully hydrolyzed with 1M HCl (20 mL), diluted with H_2_O and extracted three times with CH_2_Cl_2_. The organic phase was washed with brine, dried over Na_2_SO_4_, and then evaporated under reduced pressure. The residue was purified by flash chromatography on silica gel eluted with *V*_chloroform_:*V*_methanol_ = 30:1 to afford alcohol **7**. Yield 76%, white solid, m.p. 204–206 °C; ^1^H NMR (400 MHz, CDCl_3_) ^1^H NMR (600 MHz, CD_3_OD) *δ* 7.95 (s, 1H), 7.87 (d, *J* = 1.9 Hz, 1H), 7.55 (s, 1H), 6.97 (d, *J* = 2.0 Hz, 1H), 6.56 (s, 1H), 4.94 (s, 2H). ^13^C NMR (101 MHz, CD_3_OD) *δ* 163.57, 158.32, 152.82, 148.71, 129.23, 127.39, 117.21, 115.30, 110.23, 107.75, 100.51, 61.09.

#### 3.1.7. Preparation of (7-Oxo-*7H*-furo[3,2-g]chromen-5-yl)methyl acetate (**7**)

A mixture of **7** (0.022 g, 0.09 mmol), 4-dimethylaminopyridine DMAP (1 mg), and one drop of acetic anhydride in 2 mL of anhydrous pyridine was stirred at room temperature overnight under nitrogen atmosphere, 5 mL of icy water was then added. The reaction mixture was extracted with ethyl acetate three times, and the organic layer was washed with brine and dried over anhydrous Na_2_SO_4_. After the removal of solvent, the crude product was purified by flash column chromatography over silica gel eluted with *V*_petroleumether_/*V*_ethylacetate_ = 3:1 to give acetate **7**. Yield 80%, white solid, m.p. 198–200 °C; ^1^H NMR (400 MHz, CDCl_3_) *δ* 7.82 (s, 1H), 7.71 (d, *J* = 2.1 Hz, 1H), 7.47 (s, 1H), 6.86 (d, *J* = 2.0 Hz, 1H), 6.49 (s, 1H), 5.37 (s, 2H), 2.23 (s, 3H).

#### 3.1.8. General Procedure Of preparation of Esters **8a**–**8p**

1,3-Dicyclohexylcarbodiimide (DCC, 0.82 g, 4 mmol) was added to a solution of **7** (0.86 g, 4 mmol), different benzoic acid (5 mmol) and DMAP (0.49 g, 4 mmol) in dry CH_2_Cl_2_ (25 mL) at 0 °C under nitrogen. After 10 min at 0 °C, the mixture was stirred at room temperature for 12 h. After filtration, the organic filtrate was washed with 1.2 M hydrochloric acid, saturated aqueous sodium hydrogen carbonate, then dried over sodium sulfate and evaporated under reduced pressure. The residue was purified by flash chromatography on silica gel eluted with petroleum etherethyl acetate to give corresponding benzoate **8a**–**8p**.

(7-Oxo-*7H*-furo[3,2-g]chromen-5-yl)methyl-4-methylbenzoate (**8a**). Yield 81%, white solid, m.p. 181–182 °C; ^1^H NMR (600 MHz, CDCl_3_) *δ* 8.02 (d, *J* = 8.1 Hz, 2H), 7.81 (s, 1H), 7.71 (d, *J* = 2.1 Hz, 1H), 7.53 (s, 1H), 7.29 (d, *J* = 8.0 Hz, 2H), 6.86 (d, *J* = 2.1 Hz, 1H), 6.60 (s, 1H), 5.60 (s, 2H), 2.44 (s, 3H). ^13^C NMR (101 MHz, CDCl_3_) *δ* 165.77, 160.75, 156.37, 151.71, 149.62, 147.14, 144.67, 129.89, 129.40, 126.23, 124.94, 115.46, 113.68, 111.57, 106.57, 100.37, 61.65, 21.76; IR (KBr) *v*: 2925, 1717, 1634, 1576, 1447, 1278, 1264, 1156, 1135, 874, 750 cm^−1^; HRMS (ESI) calcd for C_20_H_15_O_5_[M + H]^+^ 335.0914, found 335.0931.

(7-Oxo-*7H*-furo[3,2-g]chromen-5-yl)methyl-2-methylbenzoate (**8b**). Yield 84%, white solid, m.p. 169–171 °C; ^1^H NMR (400 MHz, CDCl_3_) *δ* 8.04 (dd, *J* = 7.8, 0.8 Hz, 1H), 7.83 (s, 1H), 7.72 (d, *J* = 2.1 Hz, 1H), 7.54 (s, 1H), 7.50–7.44 (m, 1H), 7.33–7.28 (m, 2H), 6.87 (d, *J* = 2.2 Hz, 1H), 6.59 (s, 1H), 5.60 (s, 2H), 2.65 (s, 3H). ^13^C NMR (101 MHz, CDCl_3_) *δ* 166.28, 160.68, 156.35, 151.71, 149.55, 147.11, 141.04, 132.82, 132.00, 130.76, 125.99, 124.91, 115.45, 113.69, 111.74, 106.53, 100.36, 61.61, 21.88; IR (KBr) *v*: 2926, 1723, 1632, 1577, 1449, 1278, 1263, 1155,1135, 873, 749 cm^−1^; HRMS (ESI) calcd for C_20_H_15_O_5_[M + H]^+^ 335.0914, found 335.0944.

(7-Oxo-*7H*-furo[3,2-g]chromen-5-yl)methyl-4-methoxybenzoate (**8c**). Yield 80%, white solid, m.p. 155–157 °C; ^1^H NMR (400 MHz, CDCl_3_) *δ* 8.09 (d, *J* = 8.8 Hz, 2H), 7.82 (s, 1H), 7.71 (d, *J* = 2.1 Hz, 1H), 7.54 (s, 1H), 6.97 (d, *J* = 8.9 Hz, 2H), 6.86 (d, *J* = 2.0 Hz, 1H), 6.59 (s, 1H), 5.60 (s, 2H), 3.89 (s, 3H). ^13^C NMR (101 MHz, CDCl_3_) *δ* 165.56, 160.91, 156.52, 151.87, 149.90, 147.28, 132.12, 129.20, 125.08, 121.44, 115.62, 114.11, 113.91, 111.67, 106.72, 100.51, 61.68, 55.69; IR (KBr) *v*: 2927, 1724, 1634, 1575, 1448, 1277, 1265, 1156, 1134, 872, 748 cm^−1^; HRMS (ESI) calcd for C_20_H_15_O_6_[M + H]^+^ 351.0863, found 351.0855.

(7-Oxo-*7H*-furo[3,2-g]chromen-5-yl)methyl-2-methoxybenzoate (**8d**). Yield 82%, white solid, m.p. 148–149 °C; ^1^H NMR (400 MHz, CDCl_3_) *δ* 7.92 (dd, *J* = 7.9, 1.8 Hz, 1H), 7.82 (s, 1H), 7.71 (d, *J* = 2.2 Hz, 1H), 7.57–7.50 (m, 1H), 7.06–7.00 (m, 2H), 6.86 (d, *J* = 2.2 Hz, 1H), 6.73 (s, 1H), 5.60 (s, 2H), 3.95 (s, 3H). ^13^C NMR (101 MHz, CDCl_3_) *δ* 165.56, 160.86, 159.54, 156.33, 151.72, 149.65, 147.10, 134.49, 132.20, 124.87, 120.35, 118.61, 115.50, 113.73, 112.11, 111.79, 106.57, 100.30, 61.78, 55.94; IR (KBr) *v*: 2927, 1718, 1633, 1576, 1450, 1279, 1264, 1157, 1136, 873, 760, 750 cm^−1^; HRMS (ESI) calcd for C_20_H_15_O_6_[M + H]^+^ 351.0863, found 351.0839.

(7-Oxo-*7H*-furo[3,2-g]chromen-5-yl)methyl-4-chlorobenzoate (**8e**). Yield 78%, light yellow solid, m.p. 201–202 °C; ^1^H NMR (400 MHz, CDCl_3_) *δ* 8.07 (d, *J* = 8.6 Hz, 2H), 7.80 (s, 1H), 7.72 (d, *J* = 2.3 Hz, 1H), 7.55 (s, 1H), 7.48 (d, *J* = 8.6 Hz, 2H), 6.87 (d, *J* = 2.1 Hz, 1H), 6.57 (s, 1H), 5.62 (s, 2H). ^13^C NMR (101 MHz, CDCl_3_) *δ* 164.89, 160.63, 156.42, 151.72, 149.20, 147.22, 140.40, 131.23, 129.10, 127.41, 125.00, 115.41, 113.58, 111.72, 106.56, 100.46, 62.03; IR (KBr) *v*: 1717, 1624, 1574, 1541, 1457, 1270, 1243, 1129, 1090, 669 cm^−1^; HRMS (ESI) calcd for C_19_H_12_ClO_5_[M + H]^+^ 355.0368, found 355.0390.

(7-Oxo-*7H*-furo[3,2-g]chromen-5-yl)methyl-2-chlorobenzoate (**8f**). Yield 75%, light yellow solid, m.p. 183–185 °C; ^1^H NMR (400 MHz, CDCl_3_) δ 7.95 (dd, *J* = 7.6, 1.3 Hz, 1H), 7.82 (s, 1H), 7.72 (d, *J* = 2.3 Hz, 1H), 7.54 (s, 1H), 7.53–7.48 (m, 2H), 7.41–7.35 (m, 1H), 6.87 (d, *J* = 2.2 Hz, 1H), 6.64 (s, 1H), 5.63 (s, 2H). ^13^C NMR (101 MHz, CDCl_3_) *δ* 164.92, 160.79, 156.55, 151.89, 149.04, 147.33, 134.37, 133.55, 131.94, 131.60, 128.88, 127.01, 125.11, 115.68, 113.74, 112.24, 106.71, 100.56, 62.57; IR (KBr) *v*: 1734, 1707, 1635, 1577, 1450, 1412, 1240, 1156, 1113, 1028, 746 cm^−1^; HRMS (ESI) calcd for C_19_H_12_ClO_5_[M + H]^+^ 355.0368, found 355.0387.

(7-Oxo-*7H*-furo[3,2-g]chromen-5-yl)methyl-3,4-dichlorobenzoate (**8g**). Yield 70%, light yellow solid, m.p. 198–200 °C; ^1^H NMR (400 MHz, CDCl_3_) *δ* 8.20 (d, *J* = 2.0 Hz, 1H), 7.95 (dd, *J* = 8.4, 1.9 Hz, 1H), 7.80 (s, 1H), 7.73 (d, *J* = 2.3 Hz, 1H), 7.59 (d, *J* = 8.4 Hz, 1H), 7.55 (s, 1H), 6.87 (d, *J* = 2.1 Hz, 1H), 6.55 (s, 1H), 5.63 (s, 2H). ^13^C NMR (101 MHz, CDCl_3_) *δ* 163.97, 160.54, 158.21, 153.31, 147.28, 143.32, 138.63, 136.04, 131.73, 130.90, 128.83, 127.59, 122.64, 120.23, 115.41, 111.86, 106.56, 100.50, 62.34; IR (KBr) *v*: 1716, 1633, 1620, 1568, 1513, 1447, 1361, 1250, 1089, 748 cm^−1^; HRMS (ESI) calcd for C_19_H_11_Cl_2_O_5_[M + H]^+^ 388.9978, found 388.9955.

(7-Oxo-*7H*-furo[3,2-g]chromen-5-yl)methyl-3,5-dichlorobenzoate (**8h**). Yield 71%, light yellow solid, m.p. 223–224 °C; ^1^H NMR (400 MHz, CDCl_3_) *δ* 7.98 (d, *J* = 1.9 Hz, 2H), 7.79 (s, 1H), 7.73 (d, *J* = 2.2 Hz, 1H), 7.62 (t, *J* = 1.9 Hz, 1H), 7.55 (s, 1H), 6.87 (d, *J* = 2.1 Hz, 1H), 6.54 (s, 1H), 5.63 (s, 2H). ^13^C NMR (101 MHz, CDCl_3_) *δ* 163.67, 160.63, 156.59, 151.86, 148.84, 147.41, 135.83, 133.77, 131.88, 128.96, 128.32, 125.18, 115.52, 112.04, 106.68, 100.63, 62.64; IR (KBr) *v*: 1718, 1636, 1571, 1451, 1261, 1156, 1130, 870, 748 cm^−1^; HRMS (ESI) calcd for C_19_H_11_Cl_2_O_5_[M + H]^+^ 388.9978, found 388.9967.

(7-Oxo-*7H*-furo[3,2-g]chromen-5-yl)methyl-4-fluorobenzoate (**8i**). Yield 83%, off-white solid, m.p. 177–178 °C; ^1^H NMR (400 MHz, CDCl_3_) *δ* 8.19–8.12 (m, 2H), 7.81 (s, 1H), 7.72 (d, *J* = 2.2 Hz, 1H), 7.54 (s, 1H), 7.18 (t, *J* = 8.6 Hz, 2H), 6.87 (d, *J* = 2.1 Hz, 1H), 6.57 (s, 1H), 5.62 (s, 2H). ^13^C NMR (101 MHz, CDCl_3_) *δ* 164.87, 160.78, 156.54, 151.85, 149.47, 147.35, 146.81, 132.62, 125.13, 116.20, 115.98, 115.56, 111.78, 106.69, 100.56, 62.04; IR (KBr) *v*: 1716, 1604, 1508, 1456, 1265, 1237, 1154, 849, 743 cm^−1^; HRMS (ESI) calcd for C_19_H_12_FO_5_[M + H]^+^ 339.0663, found 339.0689.

(7-Oxo-*7H*-furo[3,2-g]chromen-5-yl)methyl-2,4-difluorobenzoate (**8j**). Yield 77%, off-white solid, m.p. 185–186 °C; ^1^H NMR (400 MHz, CDCl_3_) *δ* 8.08 (td, *J* = 8.5, 6.5 Hz, 1H), 7.81 (s, 1H), 7.72 (d, *J* = 2.2 Hz, 1H), 7.54 (s, 1H), 7.04–6.90 (m, 2H), 6.87 (d, *J* = 2.0 Hz, 1H), 6.63 (s, 1H), 5.63 (s 2H). ^13^C NMR (101 MHz, CDCl_3_) *δ* 165.18, 164.55, 162.94, 160.79, 156.54, 151.85, 149.00, 147.33, 134.41, 125.10, 115.56, 113.67, 112.34, 112.12, 111.97, 106.70, 105.76, 100.56, 62.40; IR (KBr) *v*: 1718, 1615, 1507, 1452, 1259, 1125, 1090, 852, 747 cm^−1^; HRMS (ESI) calcd for C_19_H_11_F_2_O_5_[M + H]^+^ 357.0569, found 339.0545.

(7-Oxo-*7H*-furo[3,2-g]chromen-5-yl)methyl-benzoate (**8k**). Yield 85%, off-white solid, m.p. 213–215 °C; ^1^H NMR (400 MHz, CDCl_3_) δ 8.16-8.11 (m, 2H), 7.82 (s, 1H), 7.72 (d, *J* = 2.2 Hz, 1H), 7.64 (t, *J* = 7.4 Hz, 1H), 7.56–7.47 (m, 3H), 6.87 (d, *J* = 2.2 Hz, 1H), 6.61 (s, 1H), 5.63 (s, 2H). ^13^C NMR (101 MHz, CDCl) *δ* 165.87 160.85, 156.54, 151.88, 149.61, 147.32, 133.94, 131.04, 130.01, 128.85, 125.11, 115.60, 113.81, 111.79, 106.72, 100.55, 61.96; IR (KBr) *v*: 1717, 1635, 1506, 1451, 1268, 1158, 1116, 1094, 847, 745 cm^−1^; HRMS (ESI) calcd for C_19_H_13_O_5_[M + H]^+^ 321.0757, found 321.0760.

(7-Oxo-*7H*-furo[3,2-g]chromen-5-yl)methyl-4-nitrobenzoate (**8l**). Yield 70%, off-white solid, m.p. 201–203 °C; ^1^H NMR (400 MHz, CDCl_3_) *δ* 8.38–8.26 (m, 5H), 7.81 (s, 1H), 7.73 (d, *J* = 2.3 Hz, 1H), 7.56 (s, 1H), 6.87 (d, *J* = 2.1 Hz, 1H), 6.57 (s, 1H), 5.68 (s, 2H). ^13^C NMR (101 MHz, CDCl_3_) *δ* 164.07, 156.71, 151.89, 149.63, 148.77, 147.48, 135.63, 131.18, 129.03, 126.84, 124.01, 115.49, 114.44, 112.12, 106.68, 100.72, 62.78; IR (KBr) *v*: 1720, 1624, 1577, 1527, 1451, 1270, 1244, 1089 cm^−1^; HRMS (ESI) calcd for C_19_H_12_NO_7_[M + H]^+^ 366.0608, found 366.0586.

(7-Oxo-*7H*-furo[3,2-g]chromen-5-yl)methyl-4-(trifluoromethyl)benzoate (**8m**). Yield 66%, off-white solid, m.p. 219–220 °C; ^1^H NMR (400 MHz, CDCl_3_) *δ* 8.25 (d, *J* = 8.0 Hz, 2H), 7.81 (s, 1H), 7.77 (d, *J* = 8.3 Hz, 2H), 7.73 (d, *J* = 2.3 Hz, 1H), 7.56 (s, 1H), 6.87 (d, *J* = 2.2 Hz, 1H), 6.58 (s, 1H), 5.66 (s, 2H). ^13^C NMR (101 MHz, CDCl_3_) *δ* 166.84, 160.71, 156.60, 151.87, 149.06, 147.42, 137.47, 130.44, 127.12, 125.94, 125.18, 115.53, 112.03, 110.18, 106.70, 100.67, 62.48; IR (KBr) *v*: 1734, 1718, 1625, 1578, 1559, 1326, 1271, 1131 cm^−1^; HRMS (ESI) calcd for C_20_H_12_F_3_O_5_[M + H]^+^ 389.0631, found 389.0642.

(7-Oxo-*7H*-furo[3,2-g]chromen-5-yl)methyl-3-nitrobenzoate (**8n**). Yield 68%, off-white solid, m.p. 202–204 °C; ^1^H NMR (400 MHz, CDCl_3_) *δ* 8.52–8.37 (m, 4H), 7.83 (s, 1H), 7.73 (d, *J* = 2.2 Hz, 1H), 7.56 (s, 1H), 6.88 (d, *J* = 2.1 Hz, 1H), 6.56 (s, 1H), 5.69 (s, 2H). ^13^C NMR (101 MHz, CDCl_3_) *δ* 164.82, 162.22, 156.99, 156.59, 147.46, 142.58, 135.51, 133.05, 130.20, 129.53, 128.34, 125.05, 115.55, 112.20, 109.31, 106.70, 100.71, 62.77; IR (KBr) *v*: 1733, 1719, 1624, 1576, 1532, 1351, 1270, 1087 cm^−1^; HRMS (ESI) calcd for C_19_H_12_NO_7_[M + H]^+^ 366.0608, found 366.0633.

(7-Oxo-*7H*-furo[3,2-g]chromen-5-yl)methyl-3,5-dinitrobenzoate (**8o**). Yield 52%, off-white solid, m.p. 210–211 °C; ^1^H NMR (400 MHz, CDCl_3_) *δ* 9.29 (d, *J* = 1.9 Hz, 1H), 9.23 (d, *J* = 2.0 Hz, 1H), 7.83 (s, 1H), 7.74 (d, *J* = 2.2 Hz, 1H), 7.57 (s, 1H), 6.89 (d, *J* = 2.1 Hz, 1H), 6.53 (s, 1H), 5.75 (s, 2H). ^13^C NMR (101 MHz, CDCl_3_) *δ* 165.43, 161.06, 156.74, 152.15, 148.91, 146.64, 144.84, 135.53, 132.04, 129.76, 128.71, 124.84, 116.13, 112.15, 107.32, 100.31, 62.46; IR (KBr) *v*: 1718, 1623, 1575, 1540, 1436, 1311, 1243, 1089 cm^−1^; HRMS (ESI) calcd for C_19_H_11_N_2_O_9_[M + H]^+^ 411.0459, found 411.0476.

(7-Oxo-*7H*-furo[3,2-g]chromen-5-yl)methyl-2-iodobenzoate (**8p**). Yield 78%, yellow solid, m.p. 186–187 °C; ^1^H NMR (400 MHz, CDCl_3_) *δ* 8.06 (d, *J* = 8.0 Hz, 1H), 7.92 (d, *J* = 7.6 Hz, 1H), 7.82 (s, 1H), 7.72 (d, *J* = 1.9 Hz, 1H), 7.54 (s, 1H), 7.50–7.42 (m, 2H), 6.87 (d, *J* = 2.0 Hz, 1H), 6.62 (s, 1H), 5.63 (s, 2H). ^13^C NMR (101 MHz, CDCl_3_) *δ* 160.79, 156.46, 149.03, 147.35, 141.89, 133.56, 131.41, 128.33, 125.13, 123.26, 116.70, 115.69, 113.65, 112.28, 110.17, 106.72, 100.58, 62.59; IR (KBr) *v*: 1735, 1705, 1636, 1564, 1516, 1441, 1230, 1282, 1149, 1123, 748 cm^−1^; HRMS (ESI) calcd for C_19_H_12_IO_5_[M + H]^+^ 446.9724, found 446.9741.

### 3.2. Biological Activity

Akt (also named PKB, protein kinase B), p-Akt (Ser308), GSK3β, p-GSK3β (Ser9), and β-actin antibodies were purchased from Cell Signaling Technology (Danvers, MA, USA). β-catenin, p-β-catenin (Ser33), antibodies against TYR, TRP1, and TRP2 were bought from Santa Cruz Technology (Dallas, TX, USA). Anti-MITF antibody was purchased from Millipore (Billerica, MA, USA). Anti-mouse, anti-goat and anti-rabbit IgG antibodies (horseradish peroxidase conjugated) were purchased from Santa Cruz Biotechnology (Dallas, TX, USA).

Dimethylsulfoxide (DMSO) was bought from Sigma (St. Louis, MO, USA), [2-(2-methoxy-4-nitrophenyl)-3-(4-nitrophenyl)-5-(2,4-disulfophenyl)-2*H*-tetrazolium] (CCK-8) was purchased from Trans Gen Biotechnology (Beijing, China). Nuclear and cytoplasmic protein extraction kit was bought from Beyotime Biotechnology, (Shanghai, China). Akt inhibitor IV was bought from EMD Biosciences, (La Jolla, CA, USA). BIO was purchased from AMQUAR Biology, (Shanghai, China).

#### 3.2.1. Cell Cultures

The murine B16 melanoma cell line was purchased from Chinese Academy of Sciences (Beijing, China). The cells were maintained in Dulbecco’s modified Eagle’s medium (DMEM, Gibco Life Technologies, Paris, France), supplemented with 10% (*v*/*v*) FBS, penicillin G (100 U/mL), and streptomycin (100 mg/mL) (Gibco-BRL, Grand Island, NY, USA) in 5% CO_2_ at 37 °C.

#### 3.2.2. Cell Morphology and Cell Viability Measurement

Cell morphology was examined under a LEICA DMI8 microscope (LEICA microsystems CMS GmbH, Wetzlar, Germany). The cell viability was assayed by adding CCK-8 solution. Generally speaking, The B16 cells were seeded in 96-well plates at a density of 1 × 10^4^ cells per well and were allowed to adhere for 24 h. The medium was replaced with medium containing **8n** diluted to the appropriate concentrations. The control cells were treated with DMSO at a final concentration of 0.1%. After 48 h, the culture medium of the cells was discarded, 10 μL of CCK-8 solution was added into each well and cells were incubated at 37 °C for another 2 h. The absorbance was measured at 450 nm using a Spectra Max M5 (Molecular Devices, San Jose, CA, USA). All of the assays were performed in triplicate. Absorbance of cells without treatment was regarded as 100% of cell survival. Cell viability was calculated using the following formula: cell viability (%) = (A_sample_/A_control_) × 100%.

#### 3.2.3. Melanin Measurement

B16 cells were seeded at a density of 2 × 10^5^ cells/well in a 6-well plate. After overnight incubation, test samples were added to individual wells, cells were incubated for 48 h, and were washed twice with ice-cold PBS (phosphate buffered solution). After cells lysed, the harvested cells were centrifuged, and the pellet was dissolved by adding 1 N NaOH, followed by incubation at 80 °C for 1 h. Each lysate (150 μL) was put in a 96-well microplate, and measured spectro-photometrically at 405 nm by a multi-plate reader. Protein concentration of each sample was determined by BCA Protein Assay Kit (Biomed, Beijing, China). Intracellular melanin amount was expressed as abs/μg protein was shown as a percentage value. The percentage value of the **8n**-treated cells was calculated with respect to the untreated cells.

#### 3.2.4. Tyrosinase Activity Assay

The assay for tyrosinase activity was carried out, as previously described [54], with a slight modification. B16 cells were seeded in a 6-well plate at a density of 2 × 10^5^ cells per well and allowed to attach for 24 h. Test samples were then added to individual wells. After a 24 h incubation, cells were washed with ice-cold PBS twice, lysed with 1% Triton X-100 solution containing 1% sodium deoxycholate for 30 min at −80 °C, each lysate was centrifuged at 12,000× *g* for 15 min to obtain the supernatant. After protein quantification and adjustment, 90 μL of the supernatant was incubated in duplicate with 10 μL of freshly prepared substrate solution (10 mM l-DOPA) in a well of a 96-well plate. Then the cells were incubated at 37 °C in dark for 60 min, the absorbance was measured at 490 nm and the **8n**-treated cells was presented as percentage against the untreated cells.

#### 3.2.5. Western Blot Analysis

B16 cells were treated with different concentrations of **8n** in a 6-well plate for 48 h. Cells were then lysed in cold RIPA (radio immunoprecipitation assay) Lysis Buffer (pH 7.4) containing protease and protease inhibitor cocktail [1 M 4-nitrophenyl phosphate disodium salt hexahydrate (PNPP), 1 M sodium fluoride (NaF), 10mMphenylmethanesulfonylfluoride (PMSF), 100 mM benzamidine, 100 mM *DL*-Dithiothreitol (DTT), 200mM sodium orthovanadate (OV)] for 30 min on ice. The lysates were centrifuged at 12,000 rpm for 20 min at 4 °C before the supernatant was collected. The protein samples concentration was measured by BCA Protein Assay Kit (Biomed, Beijing, China) and was separated by 10% SDS polyacrylamide gels and transferred onto polyvinylidene fluoride (PVDF) membranes (Merck Millipore Ltd., Billerica, MA, USA), Membranes were incubated with the primary antibodies at 4 °C overnight, and then incubated with horseradish peroxidase-conjugated secondary antibodies for 1 h at room temperature. The targeted proteins were detected by ECL western blotting detection reagents (GE Healthcare, Beijing, China), and were visualized using the ChemiDoc MP Imaging system (Bio-Rad Laboratories, Inc., Berkeley, CA, USA). All western bolt assay results were performed in triplicate.

#### 3.2.6. Statistical Analysis

All data were expressed as the means ± standard deviations and statistical analysis was performed by one-way ANOVA followed by Tukey post hoc test for multiple comparison tests. A *p* value of < 0.05 was considered a significant difference.

## 4. Conclusions

In summary, a series of ester furocoumarin derivatives had been prepared via total synthesis or structural modification. Half of compounds exhibited a better activity on melanin synthesis than positive control (8-MOP) in B16 melanoma cells. Among them, compounds **8n** (200%) and **8o** (197%) were nearly 1.5-fold stronger than 8-MOP (136%).

Western blot analysis showed that **8n** promoted phosphorylation of Akt, and thus probably increased the non-activated GSK3β, which inhibits the phosphorylation of β-catenin and ceased its degradation via proteasome. The accumulation of β-catenin probably leads to the activation of transcription of MITF, TYR family, and thus the subsequent induction of melanin synthesis. These results indicated that **8n** stimulated melanin biosynthesis by up-regulation of MITF and TYR family via Akt/GSK3β/β-catenin signaling pathways.

In the view of the widespread application of furocoumarins in vitiligo nowadays, similar structural modification on C-5 position may be beneficial to explore potential melanogenesis-modulating candidates. Signaling pathway research would shed light on the molecular function of furocoumarins in melanogenesis. Further studies in vivo on vitiligo model mice are under way to assess the safety and efficacy of **8n** for clinical use.
